# Factors associated with adverse drug reactions or death in very elderly hospitalized patients with pulmonary tuberculosis

**DOI:** 10.1038/s41598-023-33967-6

**Published:** 2023-04-26

**Authors:** Mitsuaki Yagi, Yuichiro Shindo, Yoshikazu Mutoh, Masahiro Sano, Toshihiro Sakakibara, Hironori Kobayashi, Akinobu Matsuura, Ryo Emoto, Shigeyuki Matsui, Taku Nakagawa, Kenji Ogawa

**Affiliations:** 1 Department of Respiratory Medicine, Nagoya University Graduate School of Medicine, 65 Tsurumai-cho, Showa-ku, Nagoya, 466-8550 Japan; 2 Department of Respiratory Medicine, National Hospital Organization Higashinagoya National Hospital, Nagoya, Japan; 3 Department of Infectious Diseases, Tosei General Hospital, Seto, Japan; 4 Department of Biostatistics, Nagoya University Graduate School of Medicine, Nagoya, Japan

**Keywords:** Outcomes research, Infectious diseases, Tuberculosis, Risk factors

## Abstract

The aging of patients with tuberculosis and better therapeutic management for them are recent concerns. This study aimed to identify risk factors for adverse drug reactions (ADRs) or death in very elderly patients with pulmonary tuberculosis and to assess the association between the dosage of antituberculosis drugs and outcomes. We conducted a multicenter retrospective study at two hospitals. Hospitalized patients (≥ 80 years old) with pulmonary tuberculosis who were treated with antituberculosis drugs were enrolled. Multivariate analysis was performed to assess factors associated with ADRs or death within 60 days after treatment initiation. In total, 632 patients were included. The primary endpoint occurred in 268 patients (190 ADRs and 78 deaths). A serum albumin level < 2.5 g/dL, respiratory failure, and dependent activities of daily living were independent risk factors for ADRs or death. However, a low dosage (< 8 mg/kg/day) of rifampicin was associated with a lower risk of the primary outcomes. Delayed time to negative sputum culture conversion was not observed in the lower dosage of rifampicin group. Very elderly hospitalized tuberculosis patients with the aforementioned risk factors should be carefully monitored to receive safer treatment. Rifampicin dosage reduction may be considered for very elderly tuberculosis patients to prevent ADRs/death.

## Introduction

Tuberculosis (TB) is one of the three major infectious diseases worldwide^1^. In Japan, although the incidence of TB is decreasing yearly, the TB incidence remains moderate in the country, while other industrialized countries have a low incidence^2,3^. One of the reasons that Japan still has a moderate incidence of TB is population aging^2^. Several countries are facing a similar issue of the aging of patients with TB^4–6^. The high incidence of tuberculosis after World War II resulted in a higher prevalence of latent tuberculosis infection (LTBI) in the current very elderly population^7^. Immunosenescence predisposes individuals to reactivation of LTBI^8,9^, and this may result in an increasing incidence rate of active TB among very elderly individuals. The proportion of very elderly individuals (aged 80 years or older) among those with active TB has steadily risen from 26.6% in 2008 to 41.7% in 2019^9^. Since the mortality rate among very elderly patients with TB is high^2^, the development of effective treatment strategies for this age group is an important challenge.

Polypharmacy related to complex comorbid diseases and age-related physiological deterioration are common among very elderly individuals^10^. Therefore, very elderly patients with TB are more likely to experience antituberculosis drug-induced adverse reactions^11^. Adverse drug reactions (ADRs) to treatment have been suggested as a possible factor that contributes to poor outcomes in elderly individuals^12^. In fact, an association between drug-induced liver injury and death has been reported^13^. In this context, treatment guidelines for TB from the United States and Japan state that dosage adjustment should be considered in the elderly population due to concerns about ADRs^14,15^. We speculate that dosage reduction of antituberculosis drugs is often attempted for those patients in clinical practice. However, evidence on the validity is scarce. Furthermore, which drugs should be reduced and in which patients remain unclear.

Therefore, it is important for physicians to recognize patients at a high risk of adverse events, including death, to provide effective and safer treatment for very elderly patients with TB. The aim of this study was to identify risk factors for the composite outcome (ADRs or death) in those patients and to assess the association between the dosage of antituberculosis drugs and the composite outcome.

## Methods

### Study design and patient population

We conducted a multicenter retrospective study of very elderly patients (aged 80 years or older) with active TB who were admitted to one of two hospitals with TB wards (Higashinagoya National Hospital and Tosei General Hospital, Japan) between January 1, 2013, and December 31, 2018. Patients with pulmonary TB who were treated with antituberculosis drugs, including isoniazid (INH) and rifampicin (RIF), at the study institutions were eligible. The exclusion criteria were as follows: infection due to strains of *Mycobacterium tuberculosis* resistant to either INH or RIF, or both; admission for desensitization therapy or other purposes due to an adverse event of antituberculosis drugs; extrapulmonary TB alone; treatment of TB was already started at other hospitals or outpatient clinic before admission; and unsuitability for the study, as determined by the researchers.

The protocol for this study adhered to the Declaration of Helsinki and the Japanese Ethics Guidelines for Epidemiological Studies. This study was approved by the institutional review boards at two hospitals. Informed consent from the participants was waived since this study was based on a retrospective review of their records and images. However, the study information was provided to the eligible patients through the internet, and they were given the opportunity to withdraw from the study.

### Data collection and definition of variables

The following data on admission were collected by chart review: demographic information, comorbidities, concomitant medications, functional status, smoking history, and physical, laboratory, and radiological findings. Details of the disease status of TB and antituberculosis treatment were also collected. Polypharmacy was defined as the use of 7 or more medications in addition to antituberculosis drugs^16^. Activities of daily living (ADL) were categorized into two groups: independent and dependent (including assisted walking, wheelchair use, and bedridden). Comorbidities included the following: chronic lung diseases, chronic heart diseases, chronic liver diseases, chronic kidney diseases, neurological disorders, active malignancy, and diabetes mellitus. Details of these definitions of comorbidities are shown in the Supplementary Information. The disease status of TB was evaluated by cavitation and the extent of shadows on radiological findings and the sputum smear grade (0, ± , 1+, 2+, and 3+). Extensive disease of TB was defined when the extent of the shadows was beyond one lung.

### Endpoint

The primary endpoint of this study was the proportion of patients who reached the composite outcome, including ADRs or death, within 60 days after TB treatment initiation. As secondary endpoints, all-cause deaths and ADRs within 60 days were separately assessed. ADRs were defined as any event that resulted in discontinuation of at least one antituberculosis drug or alteration of the dosage. The likelihood of ADRs associated with antituberculosis drugs was categorized as definite, probable, possible or unlikely, according to a previous study and WHO criteria^17,18^. We assessed ADRs using the following five criteria: (1) known adverse drug reaction; (2) temporal relationship; (3) adverse reaction disappeared with dosage reduction or discontinuation of the study medication; (4) symptoms could not be explained by any other known condition or predisposition of the patient; and (5) the symptoms reappeared upon rechallenge or laboratory tests showed higher than normal drug levels or metabolic disturbances that explained the symptoms^17^. The level of association was classified into four categories: (1) definite, all five criteria were satisfied; (2) probable, the first four criteria were satisfied; (3) possible, the first two criteria were satisfied; and (4) unlikely, relevant information could not be obtained, time relationship between ADRs and drug intake was improbable, or other conditions were considered to be the cause of the symptoms. In this study, events evaluated to be definite, probable, or possible were considered ADRs. Two pulmonologists independently assessed causality (MY and MS for cases at Higashinagoya National Hospital, and MY and MS for those at Tosei General Hospital). In cases in which either of the evaluators made an assessment of “unlikely” and the assessment was discordant between the evaluators, the case was discussed, and a final consensus was reached.

### Statistical analysis

All baseline data are presented as categorical variables. The dosage of RIF or INH per body weight per day is presented as the median and interquartile range. A chi-square test or the Mann–Whitney U test was used to compare the two groups.

Multivariate logistic regression was performed to assess factors associated with the composite outcomes of ADRs or death within 60 days after treatment initiation. The 17 candidate variables were determined with reference to previous reports (Supplementary Table S1)^4,17,19–25^, and we also added the dosages of RIF and INH per body weight. The cutoff values were determined based on previous reports and histograms of the variables^16,26–28^. A low dosage of RIF or INH was defined by taking into account the lower limits of the dosages recommended by the international guidelines (RIF: 8–12 mg/kg/day; and INH: 4–6 mg/kg/day)^28^. Regarding sample size calculation, as a rough guideline, we considered that at least 200 events (composite outcomes of ADRs or death) were needed to analyze the risk factors, taking into account approximately 20 candidate variables^29,30^. Based on our preliminary investigation, the estimated proportion of the occurrence of the composite outcome was approximately 33%. Thus, at least 600 elderly patients with TB were needed for the main analysis. However, considering that the number of candidate variables was relatively large and there was a concern about instability in the effect estimation of multivariate logistic regression analysis, a shrinkage method by L2-penalized logistic regression (ridge regression) was adopted, and odds ratios (ORs) and approximate 95% confidence intervals (CIs) were calculated^31^. With a tenfold cross-validation, we determined the penalty parameter as a minimizer of the mean deviance (minus twice the log-likelihood on the left-out data). The 95% CIs for the ORs were calculated by repeated penalized logistic regressions, including the determination of the penalty parameter, of 1000 bootstrap samples that were randomly drawn with replacement from all patients.

Factors associated with the 60-day all-cause death were also assessed by multivariate logistic regression analysis using the same variables that were used to assess factors associated with the 60-day composite outcomes of ADRs or death. Similarly, factors associated with ADRs within 60 days after treatment initiation of TB assessed by multivariate logistic regression analysis using the above same variables in patients excluding those who died before ADR occurrence. The cumulative incidence of the composite outcomes within 60 days after treatment initiation of TB and negative sputum culture conversion in the no event group were estimated using Kaplan–Meier curves. The log-rank test was performed for comparison of negative sputum culture conversion rates between different groups. Statistical data processing was performed using SPSS Statistics (version 28; IBM, Armonk, NY, USA) and R (ver. 3.6.3; R Foundation for Statistical Computing, Vienna, Austria).

## Results

### Patient characteristics

**Figure 1:**
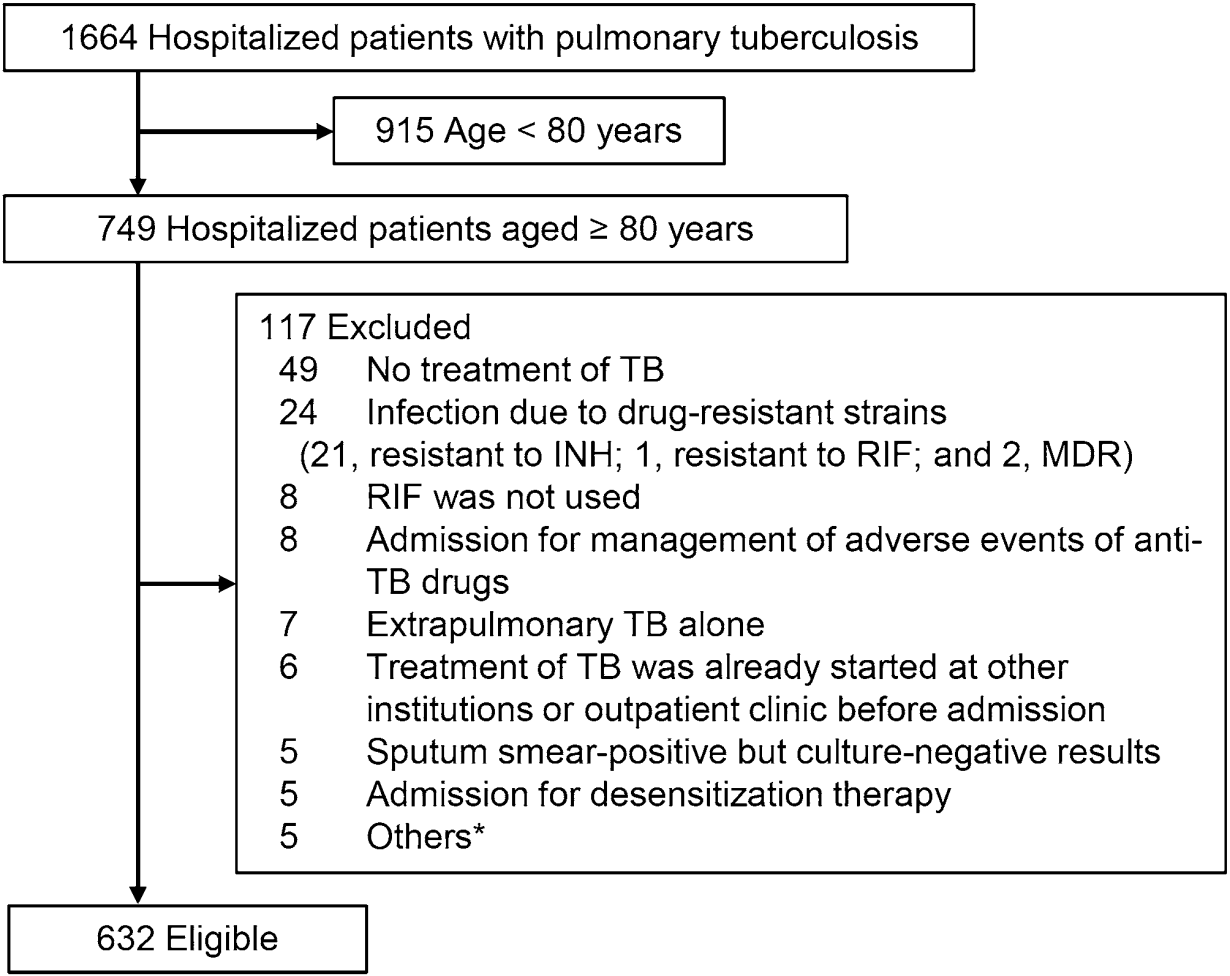
Patient flow. INH = isoniazid; RIF = rifampicin; MDR = multidrug resistance; TB = tuberculosis. *Others include 2, short-term admission to confirm adverse events; 2, clinically diagnosed cases with no bacteriological confirmation of TB; and 1, patient discharged for personal reasons.

During the study period, 1664 patients with TB were admitted to the two hospitals. Figure 1 presents the patient flow. Of the 1664 patients, 749 (45.0%) were over 80 years old. We excluded 117 patients due to no treatment (49), drug resistance (24), and other reasons that are described in Fig. 1. In total, 632 patients were eligible for this study. The baseline characteristics are shown in Table 1. Among the 632 study patients, 332 (52.5%) had low body mass index (BMI), 150 (23.7%) had polypharmacy, 468 (74.1%) had impaired ADL, and 472 (74.7%) had one or more comorbidities. Dependent ADL, extrapulmonary TB and/or extensive disease, respiratory failure, one or more comorbidities, albumin < 2.5 g/dL, and creatinine clearance < 30 mL/min were more common among patients who had ADRs or died within 60 days than among those who did not. There were no missing values.

**Table 1:** Baseline characteristics.

Variables	All patients	No event	ADR or death within 60 days	*p* value
	(n = 632)	(n = 364)	(n = 268)	
Age > 85 years	336 (53.2)	182 (50.0)	154 (57.5)	0.063
Female	251 (39.7)	147 (40.4)	104 (38.8)	0.689
BMI < 18.5 m^2^/kg	332 (52.5)	180 (49.5)	152 (56.7)	0.071
Polypharmacy^a^	150 (23.7)	87 (23.9)	63 (23.5)	0.908
Immunosuppressants^b^	25 (4.0)	14 (3.8)	11 (4.1)	0.869
ADL, dependent	468 (74.1)	241 (66.2)	227 (84.7)	< 0.001
Smoking history	267 (42.2)	156 (42.9)	111 (41.4)	0.717
Disease status				
Cavitation on chest X-ray	182 (28.8)	100 (27.5)	82 (30.6)	0.391
Extrapulmonary TB and/or extensive disease	267 (42.2)	132 (36.3)	135 (50.4)	< 0.001
Sputum smear ≥ 2 +	235 (37.2)	129 (35.4)	106 (39.6)	0.290
Respiratory failure	166 (26.3)	65 (17.9)	101 (37.7)	< 0.001
Comorbidities				
At least 1 comorbidity	472 (74.7)	256 (70.3)	216 (80.6)	0.003
Chronic lung diseases	132 (20.9)	68 (18.7)	64 (23.9)	0.112
Chronic heart diseases	170 (26.9)	91 (25.0)	79 (29.5)	0.210
Chronic liver diseases	21 (3.3)	12 (3.3)	9 (3.4)	0.966
Chronic renal diseases	76 (12.0)	45 (12.4)	31 (11.6)	0.761
Neurological disorders	216 (34.2)	125 (34.3)	91 (34.0)	0.920
Active malignancy	59 (9.3)	30 (8.2)	29 (10.8)	0.271
Diabetes mellitus	148 (23.4)	84 (23.1)	64 (23.9)	0.814
Autoimmune diseases	32 (5.1)	20 (5.5)	12 (4.5)	0.564
HIV positive	0 (0)	0 (0)	0 (0)	–
Laboratory findings				
Albumin < 2.5 g/dL	195 (30.9)	77 (21.2)	118 (44.0)	< 0.001
Abnormal AST and/or ALT^c^	160 (25.3)	84 (23.1)	76 (28.4)	0.131
Renal failure (CCr < 30 mL/min)	202 (32.0)	105 (28.8)	97 (36.2)	0.050

BMI, body mass index; ADL, activities of daily living; TB, tuberculosis; HIV, human immunodeficiency virus; AST, aspartate aminotransferase; ALT, alanine aminotransferase; CCr, creatinine clearance.

Data are presented as numbers (percentages).

^a^Concomitant drugs ≥ 7.

^b^Immunosuppressive drugs within the previous 30 days and/or corticosteroids in daily doses of at least 10 mg/day of a prednisone equivalent for more than two weeks.

^c^Above the upper limit of normal range before treatment initiation of TB.

### Treatment of TB

As shown in Table 2, 89.7% of the study patients were treated with 3-drug therapy including INH and RIF. The percentage of patients treated with pyrazinamide (PZA)-containing regimens was low (7.4%). The dosage of INH per body weight per day between patients with no events and those with ADRs or death within 60 days was similar (median dosage: 5.0 vs. 5.1), and these dosages were recommended by the guidelines^14^. On the other hand, the dosage of RIF per body weight per day in both event groups was lower than the recommended dosage in the guidelines^14^. However, the dosage was higher among patients with ADRs or death within 60 days than among those without. The median (interquartile range) RIF dosage in patients who received < 8 mg/kg/day RIF was 6.4 (5.8–7.1).

**Table 2:** Antituberculosis drug regimens and dosages.

	All patients	No event	ADR or death within 60 days	*p* value
	(n = 632)	(n = 364)	(n = 268)	
Antituberculosis drug regimens	632	364	268	
HREZ	40 (6.3)	30 (8.2)	10 (3.7)	
HRLZ	7 (1.1)	4 (1.1)	3 (1.1)	
HRE	441 (69.8)	268 (73.6)	173 (64.6)	
HRL	126 (19.9)	55 (15.1)	71 (26.5)	
HR	18 (2.8)	7 (1.9)	11 (4.1)	
Dose of rifampicin and isoniazid				
Isoniazid dose/BW, mg/kg/day	5.1 (4.5–5.7)	5.0 (4.5–5.7)	5.1 (4.5–5.7)	0.673
Isoniazid < 4 mg/kg/day	51 (8.1)^a^	35 (9.6)^a^	16 (6.0)^a^	0.096
Rifampicin dose/BW, mg/kg/day	7.1 (6.1–8.6)	7.0 (6.0–8.4)	7.5 (6.4–9.0)	0.001
Rifampicin < 8 mg/kg/day	416 (65.8)^b^	255 (70.1)^b^	161 (60.1)^b^	0.009

HREZ, isoniazid, rifampicin, ethambutol, pyrazinamide; HRLZ, isoniazid, rifampicin, levofloxacin, pyrazinamide; HRE, isoniazid, rifampicin, ethambutol; HRL, isoniazid, rifampicin, levofloxacin; HR, isoniazid, rifampicin; ADR, adverse drug reactions; BW, body weight.

Data are presented as the number (percent) or median (interquartile range).

^a^Median (interquartile range) values of INH dosage in all patients, those with no event, and those with ADR or death within 60 days were 3.7 (3.5–3.9), 3.6 (3.4–3.8), and 3.7 (3.6–3.9), respectively.

^b^Median (interquartile range) values of RIF dosage in all patients, those with no event, and those with ADR or death within 60 days were 6.4 (5.8–7.1), 6.4 (5.8–7.0), and 6.5 (5.9–7.1), respectively.

### Sixty-day outcomes

**Figure 2:**
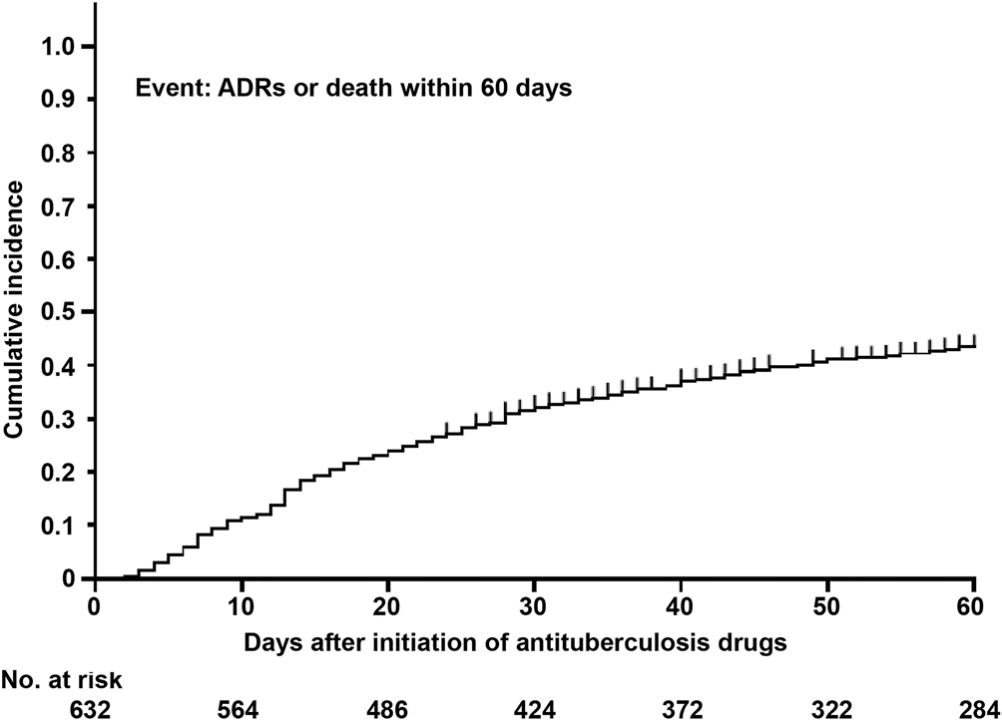
The estimate of the cumulative incidence of adverse drug reactions or death within 60 days. Vertical lines indicate censored cases that involved patients who survived and were discharged. ADR = adverse drug reaction.

Table 3 presents the 60-day outcomes. The primary endpoint, the composite outcome of death or ADRs within 60 days after treatment initiation, occurred in 268 patients (42.4%). Most events (204/268 [76.1%]) occurred within 30 days after initiation of TB treatment (Fig. 2). Of the 268 patients who reached the primary endpoint, 78 had drug discontinuation due to death (before ADR occurrence), and 190 had ADRs (Table 3). The most common ADR was gastrointestinal dysfunction (68 [35.8%] of the 190 patients with ADRs), followed by hepatitis (55/190 [28.9%]) and skin rash (31/190 [16.3%]). Ninety-four patients (14.9%) died from all causes (including ADRs) within 60 days after treatment initiation. TB was considered the cause of death in 68 (72.3%) of them.

**Table 3:** Sixty-day outcomes.

Outcomes	All patients
	(n = 632)
60-day composite outcome	268 (42.4)
Death from any cause (as events before adverse drug reaction occurrence)	78 (12.3)
TB-related death	58 (9.2)
Non-TB-related death^a^	20 (3.2)
Adverse drug reactions	190 (30.1)
Gastrointestinal dysfunction^b^	68 (10.8)
Hepatitis	55 (8.7)
Rash	31 (4.9)
Fever	11 (1.7)
Acute kidney injury	7 (1.1)
Thrombocytopenia	5 (0.8)
Pneumonia	3 (0.5)
Visual disturbance	3 (0.5)
CNS disorder	2 (0.3)
Peripheral neuropathy	2 (0.3)
Others^c^	3 (0.5)
All-cause death within 60 days^d^	94 (14.9)
TB-related	68 (10.8)
Non-TB-related	26 (4.1)

Data are presented as number (percent).

TB, tuberculosis; CNS, central nervous system.

^a^5, pneumonia; 4, heart failure; 2, lung cancer; 2, multiple organ failure; 2, senility; 1, interstitial pneumonia; 1, colorectal cancer; 1, acute myocardial infarction; 1, panperitonitis; and 1, multiple myeloma.

^b^40, anorexia; 18, gastrointestinal upset; 8, vomiting; and 2, nausea.

^c^Others were 1, hyponatremia; 1, leukopenia; and 1, eosinophilia.

^d^Patients had adverse drug reactions that occurred before death were included.

### Factors associated with the 60-day composite outcome

The findings of multivariate logistic regression analysis for the 60-day composite outcome, including ADRs or death, are shown in Table 4. The following host factors significantly increased the risk of the composite outcome: albumin < 2.5 g/dL (adjusted OR [aOR]: 1.59; 95% CI 1.29–2.29), respiratory failure (aOR: 1.51; 95% CI 1.20–2.11), and dependent ADL (aOR: 1.46; 95% CI 1.18–2.12). Moreover, regarding the types and dosages of TB drugs, a lower dosage of RIF (< 8 mg/kg/day) significantly reduced the risk of the 60-day composite outcome (aOR: 0.80; 95% CI 0.57–0.98).

**Table 4:** Multivariate analysis of risk factors for adverse drug reactions or death within 60 days.

Variables	ADRs or death		Univariate analysis	Multivariate analysis
	Yes	No	OR (95% CI)	Adjusted OR (95% CI)
Age > 85 years				
No (n = 296)	114	182	1 (ref)	1 (ref)
Yes (n = 336)	154	182	1.35 (0.98–1.86)	1.19 (0.97–1.59)
Female				
No (n = 381)	164	217	1 (ref)	1 (ref)
Yes (n = 251)	104	147	0.94 (0.68–1.29)	0.86 (0.61–1.04)
BMI < 18.5 kg/m^2^				
No (n = 300)	116	184	1 (ref)	1 (ref)
Yes (n = 332)	152	180	1.34 (0.98–1.84)	0.99 (0.74–1.20)
Polypharmacy^a^				
No (n = 482)	205	277	1 (ref)	1 (ref)
Yes (n = 150)	63	87	0.98 (0.68–1.42)	1.05 (0.80–1.43)
ADL, dependent				
No (n = 164)	41	123	1 (ref)	1 (ref)
Yes (n = 468)	227	241	2.83 (1.90–4.20)	1.46 (1.18–2.12)
Smoking history				
No (n = 365)	157	208	1 (ref)	1 (ref)
Yes (n = 267)	111	156	0.94 (0.69–1.30)	1.04 (0.83–1.35)
Extrapulmonary TB and/or extensive disease				
No (n = 365)	133	232	1 (ref)	1 (ref)
Yes (n = 267)	135	132	1.78 (1.29–2.46)	1.22 (0.98–1.59)
Sputum smear ≥ 2+				
No (n = 397)	162	235	1 (ref)	1 (ref)
Yes (n = 235)	106	129	1.19 (0.86–1.65)	1.03 (0.80–1.30)
Respiratory failure				
No (n = 466)	167	299	1 (ref)	1 (ref)
Yes (n = 166)	101	65	2.78 (1.93–4.01)	1.51 (1.20–2.11)
Chronic lung diseases				
No (n = 500)	204	296	1 (ref)	1 (ref)
Yes (n = 132)	64	68	1.37 (0.93–2.01)	1.19 (0.92–1.69)
Chronic heart diseases				
No (n = 462)	189	273	1 (ref)	1 (ref)
Yes (n = 170)	79	91	1.25 (0.88–1.79)	1.10 (0.83–1.42)
Active malignancy				
No (n = 573)	239	334	1 (ref)	1 (ref)
Yes (n = 59)	29	30	1.35 (0.79–2.31)	1.18 (0.81–1.79)
Diabetes mellitus				
No (n = 484)	204	280	1 (ref)	1 (ref)
Yes (n = 148)	64	84	1.05 (0.72–1.52)	1.02 (0.77–1.32)
Albumin < 2.5 g/dL				
No (n = 437)	150	287	1 (ref)	1 (ref)
Yes (n = 195)	118	77	2.93 (2.07–4.16)	1.59 (1.29–2.29)
Abnormal baseline AST and/or ALT				
No (n = 472)	192	280	1 (ref)	1 (ref)
Yes (n = 160)	76	84	1.32 (0.92–1.89)	1.02 (0.76–1.32)
Renal failure (CCr < 30 mL/min)				
No (n = 430)	171	259	1 (ref)	1 (ref)
Yes (n = 202)	97	105	1.40 (1.00–1.96)	1.16 (0.91–1.55)
RIF < 8 mg/kg/day				
No (n = 216)	107	109	1 (ref)	1 (ref)
Yes (n = 416)	161	255	0.64 (0.46–0.90)	0.80 (0.57–0.98)
INH < 4 mg/kg/day				
No (n = 581)	252	329	1 (ref)	1 (ref)
Yes (n = 51)	16	35	0.60 (0.32–1.10)	0.72 (0.37–1.05)
PZA use				
No (n = 585)	255	330	1 (ref)	1 (ref)
Yes (n = 47)	13	34	0.50 (0.26–0.96)	0.88 (0.54–1.43)

OR, odds ratio; CI, confidence interval; ref, reference; BMI, body mass index; ADL, activities of daily living; ALB, albumin; AST, aspartate aminotransferase; ALT, alanine aminotransferase; CCr, creatinine clearance; RIF, rifampicin; INH, isoniazid; PZA, pyrazinamide.

^a^Concomitant drugs ≥ 7.

### Subanalyses of factors associated with each outcome: death and ADRs within 60 days after TB treatment initiation

Factors associated with 60-day all-cause mortality were also analyzed, and the findings are shown in Supplementary Table S2. The host factors and disease statuses that significantly increased the risk of 60-day death were as follows: albumin < 2.5 g/dL (aOR: 3.01; 95% CI 2.14–5.75), respiratory failure (aOR: 2.11; 95% CI 1.40–3.62), dependent ADL (aOR: 1.98; 95% CI 1.41–4.52), active malignancy (aOR: 1.93; 95% CI 1.10–3.72), extrapulmonary TB and/or extensive disease (aOR: 1.80; 95% CI 1.34–2.96), BMI < 18.5 kg/m^2^ (aOR: 1.52; 95% CI 1.08–2.55), and renal failure (aOR: 1.44; 95% CI 1.01–2.55). A lower dosage of RIF significantly reduced the risk of 60-day mortality (aOR 0.69; 95% CI 0.43–0.97).

Furthermore, factors associated with ADRs within 60 days after treatment initiation of TB were analyzed in 554 patients, excluding 78 of 632 eligible patients who died before ADR occurrence (Supplementary Table S3). Significant risk factors of ADRs included dependent ADL (aOR: 1.15; 95% CI 1.03–1.78) and albumin < 2.5 g/dL (aOR: 1.14; 95% CI 1.01–1.79).

### Time to negative sputum culture conversion in the no event group

Supplementary Fig. S1 shows Kaplan–Meier curves that estimate the cumulative incidence of negative sputum culture conversion in patients who had no event (patients without adverse drug reactions who were alive within 60 days). The time to negative sputum culture conversion tended to be shorter in patients who received a lower dosage of RIF (< 8 mg/kg/day) than in those who received ≥ 8 mg/kg/day RIF (log-rank, *p* = 0.09). No readmissions and no relapses due to drug resistance were observed in either patient group during the observation period.

## Discussion

We identified factors that were significantly associated with ADRs or death within 60 days after the initiation of TB treatment in 632 hospitalized patients with pulmonary TB who were 80 years or older. One of the strengths of this study is that the factors were assessed using a large cohort of very elderly patients with TB from multiple institutions. In this study, hypoalbuminemia, respiratory failure, and dependent ADL significantly increased the risk of ADRs or death. However, a low dosage of RIF (< 8 mg/kg/day) decreased the risk of ADRs or death. In addition, a delayed time to negative sputum culture conversion was not observed for patients who received a low dosage of RIF.

In this study, the proportion of patients who died within 60 days was 14.9%, and the proportion of those with ADRs was 30.1%. Previous studies in the same age group reported that the proportions of patients with ADRs were between 21.4 and 30.0%^11,13,32,33^. A previous study of a Japanese cohort reported that the 60-day mortality was 19.7%^11^. Thus, the rates of ADRs and mortality in previous studies were similar to the findings of this study. These results imply that the therapeutic situation in this study might not deviate from that in other previous studies, although there seemed to be some regional differences in drug regimens^11,13,32,33^. Regarding treatment strategy in Japan, PZA had not been recommended for patients who were 80 years or older until 2018^15^. Levofloxacin was substituted for ethambutol for elderly patients who were bedridden because of the difficulty of ophthalmologic assessment. These situations reflected actual prescribed regimens in Table 2.

There are many patterns of ADRs including mild to severe events. In this study, ADRs were defined as any event that resulted in discontinuation of at least one antituberculosis drug or alteration of the dosage. In other words, defined ADRs meant higher grade of ones, not mild. We used the composite outcome of ADRs or death as the primary endpoint. Many risk factors for death and ADRs in patients with TB overlap (Supplementary Table S1). We need to take into account that ADRs tend to occur in patients at risk of death. However, when assessing the two endpoints (ADRs and death) separately, patients who died early (before ADR occurrence) were assigned to the no ADRs group, which was problematic. Thus, separate assessment of the factors associated with death and ADRs may fail to identify patients at a high risk of ADRs who need effective and safer treatment, including modification of the antituberculosis drug dosage. Furthermore, in clinical practice, very elderly patients with TB who are likely to die are often in a debilitated condition at the time of TB diagnosis. They may require drug dosage adjustment and monitoring as they are at a high risk for ADRs. Moreover, an excessive dosage of a drug may increase ADRs, whereas an insufficient dosage may decrease effectiveness of the treatment and increase mortality^34^. Therefore, to identify very elderly patients with TB who need more effective and safer treatment, we considered that combining two endpoints, ADRs and death, was a better method. Thus, we assessed factors associated with the composite endpoint rather than separately assessing ADRs and death.

The possible explanations for the association between the factors identified in this study and ADRs and death are as follows. It has been reported that hypoalbuminemia increases free drug concentrations and ADRs^35^. In fact, previous studies revealed that hypoalbuminemia was a risk factor for ADRs^20^. Moreover, hypoalbuminemia reflects poor nutritional status, and poor nutrition is associated with mortality risk^6,20^. Regarding the relationship between the decline in ADL and ADRs, a previous study by Lim et al. indicated that a decline in ADL caused a decline in gastrointestinal function, which resulted in anorexia as an ADR^36^. Moreover, previous studies reported that decreased ADL was a risk factor for drug-induced liver injury^23^ and that frailty was a risk factor for ADRs^37^. In addition, previous studies have demonstrated that a decline in ADL was associated with mortality risk^22,37^. Thus, the findings of this study were consistent with those of previous studies. Respiratory failure was thought to reflect the severity of TB and has been reported to be a risk factor for death in previous reports^19,22^. Indeed, respiratory failure was an independent risk factor for 60-day all-cause death in our study (Supplementary Table S2).

In this study, a low dosage of RIF was associated with a decreased risk for ADRs or death. On the other hand, a low dosage of INH did not significantly decrease the risk of ADRs or death. There is a concern that dosage reduction of RIF will lead to delayed negative sputum culture conversion^38^. However, a low dosage of RIF was not found to be associated with delayed negative sputum culture conversion. Current guidelines for TB treatment state that dosage adjustment for elderly patients may be necessary^14,15^. The findings of this study suggest that dosage adjustment of RIF is necessary in very elderly TB patients, particularly those with the aforementioned risk factors for ADRs or death. To the best of our knowledge, there are no data on what degree of reduction in the dosage of RIF is acceptable. The median dosage of RIF in patients who received < 8 mg/kg/day RIF was 6.5 mg/kg/day in this study. This may be helpful for planning future clinical trials. Verification of this dosage is also required along with an analysis of pharmacokinetics (PK) and pharmacodynamics (PD). The PK/PD of RIF is concentration-dependent^39,40^, as well as that of INH^41^. A previous study reported that the early bactericidal activity of RIF increased with the increasing of the dosage of RIF^40^. Thus, further investigations, including prospective interventional studies, are needed to achieve effective and safer treatment for TB, including dosage modification of TB drugs such as RIF.

This study has several limitations. First, this study was performed in a retrospective manner, and thus, potential bias could not be eliminated. Prospective studies are needed to validate the risk factors for ADRs or death within 60 days after the initiation of TB treatment that were identified in our study. Second, the usefulness of RIF dosage reduction needs to be confirmed in prospective comparative studies with a control group. In this study, RIF dosage had already been reduced in many cases at the discretion of each physician. One of the possible reasons was that physicians who had many experiences for very elderly TB patients have known by experience that reduction of RIF with taking into account their general condition led to reduce ADRs and did not affect the therapeutic efficacy. However, we recognize that a limitation of this study was that we could not assess clear reasons why RIF dosage was reduced. Therefore, when performing the future prospective comparative studies, we should adopt the clear objective criteria of the drug dosage reduction in which liver conditions as well as of interference of RIF with other drugs are considered. Finally, the patients enrolled in this study were all from Japan. Previous reports revealed that Asian race was a risk factor for ADRs to antituberculosis drugs and that racial differences in N-acetyltransferase 2 gene polymorphisms were related to INH metabolism^42,43^. Therefore, the findings of this study should be validated in patients of different races.

## Conclusions

In conclusion, we identified independent factors associated with the composite endpoint, which was ADRs and death within 60 days after treatment initiation of TB. Patients with dependent ADL, respiratory failure, and hypoalbuminemia on admission are at high risk for ADRs to antituberculosis drugs or death. Physicians should carefully monitor these patients to provide safer treatment for TB. Furthermore, dosage reduction of RIF should be considered in the treatment of very elderly hospitalized patients with pulmonary TB.

## Supplementary Information

Supplementary Information.

## Data Availability

The datasets analyzed during the current study are available from the corresponding author on reasonable request.

## Abbreviations

ADL: Activities of daily living
ADRs: Adverse drug reactions
BMI: Body mass index
CI: Confidence interval
INH: Isoniazid
LTBI: Latent tuberculosis infection
OR: Odds ratio
PD: Pharmacodynamics
PK: Pharmacokinetics
PZA: Pyrazinamide
RIF: Rifampicin
TB: Tuberculosis
