# Supplementary material for: Factors associated with adverse drug reactions or death in very elderly hospitalized patients with pulmonary tuberculosis
Source: Sci Rep. 2023 Apr 26;13:6826. doi: 10.1038/s41598-023-33967-6 (PMC10133295; doi:10.1038/s41598-023-33967-6)
Supplement: Supplementary file 1 — Supplementary Information. [file 41598_2023_33967_MOESM1_ESM.docx]

**Supplementary Information**

**Factors associated with adverse drug reactions or death in very elderly hospitalized patients with pulmonary tuberculosis**

Mitsuaki Yagi^1,2^, Yuichiro Shindo^1^, Yoshikazu Mutoh^3^, Masahiro Sano^1,2^, Toshihiro Sakakibara^1^, Hironori Kobayashi^1^, Akinobu Matsuura^1^, Ryo Emoto^4^, Shigeyuki Matsui^4^, Taku Nakagawa^2^, Kenji Ogawa^2^

1) Department of Respiratory Medicine, Nagoya University Graduate School of Medicine, Nagoya, Japan

2) Department of Respiratory Medicine, National Hospital Organization Higashinagoya National Hospital, Nagoya, Japan.

3) Department of Infectious Diseases, Tosei General Hospital, Seto, Japan

4) Department of Biostatistics, Nagoya University Graduate School of Medicine, Nagoya, Japan

**Definitions of comorbidities**

Chronic lung diseases included chronic obstructive pulmonary disease (COPD) and structural lung diseases such as bronchiectasis, silicosis, pulmonary tuberculosis sequelae, interstitial lung diseases, and lung cancer. Chronic heart diseases included chronic heart failure, ischemic heart disease, and heart diseases treated with diuretics. Chronic liver diseases included preexisting viral or toxic hepatopathy. Chronic kidney diseases were defined as preexisting renal disease, and a glomerular filtration rate less than 60 mL/min calculated using the Cockcroft-Gault equation, except for abnormal values due to tuberculosis. Neurological disorders included acute or chronic cerebrovascular diseases and nonvascular encephalopathy, such as degenerative disease (e.g., Parkinson's disease/syndrome). Active malignancy was defined as any malignancy (solid tumor or hematologic malignancy) at the time of TB diagnosis that required antineoplastic treatment within the previous five years or was untreated and under observation. Patients with diabetes mellitus included those who were receiving treatment with oral antidiabetic agents or insulin or who had ≥6.5% hemoglobin A1c at the time of tuberculosis diagnosis.

**Supplementary Table S1. Factors reported in previous studies**

| Variables | Adverse drug reactions | Death |
| --- | --- | --- |
| Sex | [1-5](#_ENREF_1) | [6-11](#_ENREF_6) |
| Age | [3](#_ENREF_3),[4](#_ENREF_4) | [7](#_ENREF_7),[11](#_ENREF_11) |
| BMI |  | [6](#_ENREF_6),[12](#_ENREF_12) |
| Polypharmacy | [5](#_ENREF_5),[13](#_ENREF_13) |  |
| ADL | [14](#_ENREF_14) | [8](#_ENREF_8),[10](#_ENREF_10) |
| Smoking | [5](#_ENREF_5) |  |
| Extrapulmonary TB and/or extensive disease | [1](#_ENREF_1),[2](#_ENREF_2) | [7](#_ENREF_7),[11](#_ENREF_11),[15](#_ENREF_15) |
| Sputum smear |  | [8](#_ENREF_8),[11](#_ENREF_11) |
| Respiratory failure |  | [6](#_ENREF_6),[10](#_ENREF_10) |
| Chronic lung diseases |  | [15](#_ENREF_15),[16](#_ENREF_16) |
| Chronic heart diseases | [13](#_ENREF_13) | [15](#_ENREF_15) |
| Active malignancy | [5](#_ENREF_5) | [11](#_ENREF_11),[15](#_ENREF_15) |
| Diabetes mellitus | [17](#_ENREF_17),[18](#_ENREF_18) | [15](#_ENREF_15),[16](#_ENREF_16) |
| Malnutrition | [1](#_ENREF_1),[2](#_ENREF_2) | [10](#_ENREF_10),[12](#_ENREF_12),[19](#_ENREF_19) |
| Abnormal baseline AST and/or ALT | [4](#_ENREF_4),[14](#_ENREF_14) |  |
| Renal failure | [13](#_ENREF_13) | [6](#_ENREF_6),[11](#_ENREF_11),[16](#_ENREF_16) |
| Dosages of RIF |  |  |
| Dosages of INH |  |  |
| Use of PZA | [4](#_ENREF_4) |  |

Abbreviations: BMI, body mass index; ADL, activities of daily living; TB, tuberculosis; AST, aspartate aminotransferase; ALT, alanine aminotransferase; RIF, rifampicin; INH, isoniazid; PZA, pyrazinamide.

Numbers indicate the following references:

1 Pande, J. N., Singh, S. P., Khilnani, G. C., Khilnani, S. & Tandon, R. K. Risk factors for hepatotoxicity from antituberculosis drugs: a case-control study. *Thorax* **51**, 132-136, doi:10.1136/thx.51.2.132 (1996).

2 Sharma, S. K., Balamurugan, A., Saha, P. K., Pandey, R. M. & Mehra, N. K. Evaluation of clinical and immunogenetic risk factors for the development of hepatotoxicity during antituberculosis treatment. *Am. J. Respir. Crit. Care Med.* **166**, 916-919, doi:10.1164/rccm.2108091 (2002).

3 Yee, D. *et al.* Incidence of serious side effects from first-line antituberculosis drugs among patients treated for active tuberculosis. *Am. J. Respir. Crit. Care Med.* **167**, 1472-1477, doi:10.1164/rccm.200206-626OC (2003).

4 Marra, F. *et al.* Adverse drug reactions associated with first-line anti-tuberculosis drug regimens. *Int. J. Tuberc. Lung Dis.* **11**, 868-875 (2007).

5 Yew, W. W., Yoshiyama, T., Leung, C. C. & Chan, D. P. Epidemiological, clinical and mechanistic perspectives of tuberculosis in older people. *Respirology* **23**, 567-575, doi:10.1111/resp.13303 (2018).

6 Rao, V. K., Iademarco, E. P., Fraser, V. J. & Kollef, M. H. The impact of comorbidity on mortality following in-hospital diagnosis of tuberculosis. *Chest* **114**, 1244-1252, doi:10.1378/chest.114.5.1244 (1998).

7 Sacks, L. V. & Pendle, S. Factors related to in-hospital deaths in patients with tuberculosis. *Arch. Intern. Med.* **158**, 1916-1922, doi:10.1001/archinte.158.17.1916 (1998).

8 de Vallière, S. & Barker, R. D. Poor performance status is associated with early death in patients with pulmonary tuberculosis. *Trans. R. Soc. Trop. Med. Hyg.* **100**, 681-686, doi:10.1016/j.trstmh.2005.09.007 (2006).

9 Salvado, M. *et al.* Mortality of tuberculosis in very old people. *J. Am. Geriatr. Soc.* **58**, 18-22, doi:10.1111/j.1532-5415.2009.02619.x (2010).

10 Horita, N. *et al.* Development and validation of a tuberculosis prognostic score for smear-positive in-patients in Japan. *Int. J. Tuberc. Lung Dis.* **17**, 54-60, doi:10.5588/ijtld.12.0476 (2013).

11 Yen, Y. F. *et al.* Determinants of mortality in elderly patients with tuberculosis: a population-based follow-up study. *Epidemiol. Infect.* **145**, 1374-1381, doi:10.1017/S0950268817000152 (2017).

12 Kim, H. J. *et al.* The impact of nutritional deficit on mortality of in-patients with pulmonary tuberculosis. *Int. J. Tuberc. Lung Dis.* **14**, 79-85 (2010).

13 Onder, G. *et al.* Development and validation of a score to assess risk of adverse drug reactions among in-hospital patients 65 years or older: the GerontoNet ADR risk score. *Arch. Intern. Med.* **170**, 1142-1148, doi:10.1001/archinternmed.2010.153 (2010).

14 Horita, N. *et al.* Decreased activities of daily living is a strong risk factor for liver injury by anti-tuberculosis drugs. *Respirology* **18**, 474-479, doi:10.1111/resp.12008 (2013).

15 Dewan, P. K. *et al.* Risk factors for death during tuberculosis treatment in Orel, Russia. *Int. J. Tuberc. Lung Dis.* **8**, 598-602 (2004).

16 Chiang, C. Y. *et al.* Tuberculosis outcomes in Taipei: factors associated with treatment interruption for 2 months and death. *Int. J. Tuberc. Lung Dis.* **13**, 105-111 (2009).

17 Lin, H. S. *et al.* The clinical outcomes of oldest old patients with tuberculosis treated by regimens containing rifampicin, isoniazid, and pyrazinamide. *Clin. Interv. Aging* **11**, 299-306, doi:10.2147/CIA.S95411 (2016).

18 Siddiqui, A. N., Khayyam, K. U. & Sharma, M. Effect of Diabetes Mellitus on Tuberculosis Treatment Outcome and Adverse Reactions in Patients Receiving Directly Observed Treatment Strategy in India: A Prospective Study. *Biomed Res Int* **2016**, 7273935, doi:10.1155/2016/7273935 (2016).

19 Matos, E. D. & Moreira Lemos, A. C. Association between serum albumin levels and in-hospital deaths due to tuberculosis. *Int. J. Tuberc. Lung Dis.* **10**, 1360-1366 (2006).

**Supplementary Table S2. Multivariate analysis of risk factors for 60-day mortality**

| Variables | | 60-day death | |  | Univariate analysis |  | Multivariate analysis |
| --- | --- | --- | --- | --- | --- | --- | --- |
|  |  | Yes | No |  | OR (95% CI) |  | Adjusted OR (95% CI) |
| Age > 85 years | |  |  |  |  |  |  |
|  | No (n = 296) | 43 | 253 |  | 1 (ref) |  | 1 (ref) |
|  | Yes (n = 336) | 51 | 285 |  | 1.05 (0.68–1.63) |  | 1.00 (0.63–1.46) |
| Female | |  |  |  |  |  |  |
|  | No (n = 381) | 58 | 323 |  | 1 (ref) |  | 1 (ref) |
|  | Yes (n = 251) | 36 | 215 |  | 0.93 (0.59–1.46) |  | 0.77 (0.43–1.08) |
| BMI < 18.5 kg/m^2^ | |  |  |  |  |  |  |
|  | No (n = 300) | 24 | 276 |  | 1 (ref) |  | 1 (ref) |
|  | Yes (n = 332) | 70 | 262 |  | 3.07 (1.88–5.03) |  | 1.52 (1.08–2.55) |
| Polypharmacy^*^ | |  |  |  |  |  |  |
|  | No (n = 482) | 78 | 404 |  | 1 (ref) |  | 1 (ref) |
|  | Yes (n = 150) | 16 | 134 |  | 0.62 (0.35–1.10) |  | 0.91 (0.53–1.49) |
| ADL, dependent | |  |  |  |  |  |  |
|  | No (n = 164) | 3 | 161 |  | 1 (ref) |  | 1 (ref) |
|  | Yes (n = 468) | 91 | 377 |  | 12.95 (4.04–41.52) |  | 1.98 (1.41–4.52) |
| Smoking history | |  |  |  |  |  |  |
|  | No (n = 365) | 56 | 309 |  | 1 (ref) |  | 1 (ref) |
|  | Yes (n = 267) | 38 | 229 |  | 0.92 (0.59–1.43) |  | 1.16 (0.82–1.88) |
| Extrapulmonary TB and/or extensive disease | |  |  |  |  |  |  |
|  | No (n = 365) | 30 | 335 |  | 1 (ref) |  | 1 (ref) |
|  | Yes (n = 267) | 64 | 203 |  | 3.52 (2.21–5.62) |  | 1.80 (1.34–2.96) |
| Sputum smear ≥ 2+ | |  |  |  |  |  |  |
|  | No (n = 397) | 53 | 344 |  | 1 (ref) |  | 1 (ref) |
|  | Yes (n = 235) | 41 | 194 |  | 1.37 (0.88–2.14) |  | 0.96 (0.59–1.39) |
| Respiratory failure | |  |  |  |  |  |  |
|  | No (n = 466) | 40 | 426 |  | 1 (ref) |  | 1 (ref) |
|  | Yes (n = 166) | 54 | 112 |  | 5.14 (3.25–8.12) |  | 2.11 (1.40–3.62) |
| Chronic lung diseases | |  |  |  |  |  |  |
|  | No (n = 500) | 70 | 430 |  | 1 (ref) |  | 1 (ref) |
|  | Yes (n = 132) | 24 | 108 |  | 1.37 (0.82–2.27) |  | 1.23 (0.76–2.15) |
| Chronic heart diseases | |  |  |  |  |  |  |
|  | No (n = 462) | 71 | 391 |  | 1 (ref) |  | 1 (ref) |
|  | Yes (n = 170) | 23 | 147 |  | 0.86 (0.52–1.43) |  | 0.83 (0.47–1.19) |
| Active malignancy | |  |  |  |  |  |  |
|  | No (n = 573) | 78 | 495 |  | 1 (ref) |  | 1 (ref) |
|  | Yes (n = 59) | 16 | 43 |  | 2.36 (1.27–4.40) |  | 1.93 (1.10–3.72) |
| Diabetes mellitus | |  |  |  |  |  |  |
|  | No (n = 484) | 68 | 416 |  | 1 (ref) |  | 1 (ref) |
|  | Yes (n = 148) | 26 | 122 |  | 1.30 (0.80–2.14) |  | 1.39 (0.94–2.42) |
| Albumin < 2.5 g/dL | |  |  |  |  |  |  |
|  | No (n = 437) | 26 | 411 |  | 1 (ref) |  | 1 (ref) |
|  | Yes (n = 195) | 68 | 127 |  | 8.46 (5.17–13.87) |  | 3.01 (2.14–5.75) |
| Abnormal baseline AST and/or ALT | |  |  |  |  |  |  |
|  | No (n = 472) | 61 | 411 |  | 1 (ref) |  | 1 (ref) |
|  | Yes (n = 160) | 33 | 127 |  | 1.75 (1.10–2.80) |  | 0.99 (0.58–1.46) |
| Renal failure (CCr < 30 mL/min) | |  |  |  |  |  |  |
|  | No (n = 430) | 55 | 375 |  | 1 (ref) |  | 1 (ref) |
|  | Yes (n = 202) | 39 | 163 |  | 1.63 (1.04–2.56) |  | 1.44 (1.01–2.55) |
| RIF < 8 mg/kg/day | |  |  |  |  |  |  |
|  | No (n = 216) | 45 | 171 |  | 1 (ref) |  | 1 (ref) |
|  | Yes (n = 416) | 49 | 367 |  | 0.51 (0.33–0.79) |  | 0.69 (0.43–0.97) |
| INH < 4 mg/kg/day | |  |  |  |  |  |  |
|  | No (n = 581) | 91 | 490 |  | 1 (ref) |  | 1 (ref) |
|  | Yes (n = 51) | 3 | 48 |  | 0.34 (0.10–1.10) |  | 0.62 (0.21–1.15) |
| PZA use | |  |  |  |  |  |  |
|  | No (n = 585) | 92 | 493 |  | 1 (ref) |  | 1 (ref) |
|  | Yes (n = 47) | 2 | 45 |  | 0.24 (0.06–1.00) |  | 0.68 (0.22–1.31) |

Abbreviations: OR, odds ratio; CI, confidence interval; ref, reference; BMI, body mass index;

ADL, activities of daily living; TB, tuberculosis; AST, aspartate aminotransferase; ALT, alanine aminotransferase; CCr, creatinine clearance; RIF, rifampicin; INH, isoniazid; PZA, pyrazinamide.

^*^Concomitant drugs ≥ 7

**Supplementary Table S3. Multivariate analysis of risk factors for adverse drug reactions within 60 days after treatment initiation of tuberculosis**

| Variables | | 60-day adverse drug reactions | |  | Univariate analysis |  | Multivariate analysis |
| --- | --- | --- | --- | --- | --- | --- | --- |
|  |  | Yes | No |  | OR (95% CI) |  | Adjusted OR (95% CI) |
| Age > 85 years | |  |  |  |  |  |  |
|  | No (n = 258) | 76 | 182 |  | 1 (ref) |  | 1 (ref) |
|  | Yes (n = 296) | 114 | 182 |  | 1.50 (1.05–2.14) |  | 1.10 (0.99–1.66) |
| Female | |  |  |  |  |  |  |
|  | No (n = 334) | 117 | 217 |  | 1 (ref) |  | 1 (ref) |
|  | Yes (n = 220) | 73 | 147 |  | 0.92 (0.64–1.32) |  | 0.96 (0.64–1.06) |
| BMI < 18.5 kg/m^2^ | |  |  |  |  |  |  |
|  | No (n = 281) | 97 | 184 |  | 1 (ref) |  | 1 (ref) |
|  | Yes (n = 273) | 93 | 180 |  | 0.98 (0.69–1.39) |  | 0.97 (0.66–1.08) |
| Polypharmacy^*^ | |  |  |  |  |  |  |
|  | No (n = 417) | 140 | 277 |  | 1 (ref) |  | 1 (ref) |
|  | Yes (n = 137) | 50 | 87 |  | 1.14 (0.76–1.70) |  | 1.04 (0.86–1.44) |
| ADL, dependent | |  |  |  |  |  |  |
|  | No (n = 162) | 39 | 123 |  | 1 (ref) |  | 1 (ref) |
|  | Yes (n = 392) | 151 | 241 |  | 1.98 (1.31–2.99) |  | 1.15 (1.03–1.78) |
| Smoking history | |  |  |  |  |  |  |
|  | No (n = 319) | 111 | 208 |  | 1 (ref) |  | 1 (ref) |
|  | Yes (n = 235) | 79 | 156 |  | 0.95 (0.67–1.35) |  | 1.00 (0.78–1.21) |
| Extrapulmonary TB and/or extensive disease | |  |  |  |  |  |  |
|  | No (n = 338) | 106 | 232 |  | 1 (ref) |  | 1 (ref) |
|  | Yes (n = 216) | 84 | 132 |  | 1.39 (0.97–1.99) |  | 1.07 (0.91–1.44) |
| Sputum smear ≥ 2+ | |  |  |  |  |  |  |
|  | No (n = 353) | 118 | 235 |  | 1 (ref) |  | 1 (ref) |
|  | Yes (n = 201) | 72 | 129 |  | 1.11 (0.77–1.60) |  | 1.02 (0.83–1.30) |
| Respiratory failure | |  |  |  |  |  |  |
|  | No (n = 437) | 138 | 299 |  | 1 (ref) |  | 1 (ref) |
|  | Yes (n = 117) | 52 | 65 |  | 1.73 (1.14–2.62) |  | 1.12 (0.96–1.65) |
| Chronic lung diseases | |  |  |  |  |  |  |
|  | No (n = 444) | 148 | 296 |  | 1 (ref) |  | 1 (ref) |
|  | Yes (n = 110) | 42 | 68 |  | 1.24 (0.80–1.90) |  | 1.06 (0.88–1.59) |
| Chronic heart diseases | |  |  |  |  |  |  |
|  | No (n = 404) | 131 | 273 |  | 1 (ref) |  | 1 (ref) |
|  | Yes (n = 150) | 59 | 91 |  | 1.35 (0.92–1.99) |  | 1.07 (0.91–1.45) |
| Active malignancy | |  |  |  |  |  |  |
|  | No (n = 507) | 173 | 334 |  | 1 (ref) |  | 1 (ref) |
|  | Yes (n = 47) | 17 | 30 |  | 0.94 (0.52–1.69) |  | 1.03 (0.73–1.64) |
| Diabetes mellitus | |  |  |  |  |  |  |
|  | No (n = 426) | 146 | 280 |  | 1 (ref) |  | 1 (ref) |
|  | Yes (n = 128) | 44 | 84 |  | 1.01 (0.66–1.52) |  | 1.00 (0.73–1.27) |
| Albumin < 2.5 g/dL | |  |  |  |  |  |  |
|  | No (n = 415) | 128 | 287 |  | 1 (ref) |  | 1 (ref) |
|  | Yes (n = 139) | 62 | 77 |  | 1.81 (1.22–2.68) |  | 1.14 (1.01–1.79) |
| Abnormal baseline AST and/or ALT | |  |  |  |  |  |  |
|  | No (n = 420) | 140 | 280 |  | 1 (ref) |  | 1 (ref) |
|  | Yes (n = 134) | 50 | 84 |  | 1.19 (0.79–1.78) |  | 1.03 (0.77–1.34) |
| Renal failure (CCr < 30 mL/min) | |  |  |  |  |  |  |
|  | No (n = 386) | 127 | 259 |  | 1 (ref) |  | 1 (ref) |
|  | Yes (n = 168) | 63 | 105 |  | 1.22 (0.84–1.79) |  | 1.04 (0.86–1.39) |
| RIF < 8 mg/kg/day | |  |  |  |  |  |  |
|  | No (n = 177) | 68 | 109 |  | 1 (ref) |  | 1 (ref) |
|  | Yes (n = 377) | 122 | 255 |  | 0.77 (0.53–1.11) |  | 0.93 (0.63–1.05) |
| INH < 4 mg/kg/day | |  |  |  |  |  |  |
|  | No (n = 506) | 177 | 329 |  | 1 (ref) |  | 1 (ref) |
|  | Yes (n = 48) | 13 | 35 |  | 0.69 (0.36–1.34) |  | 0.90 (0.42–1.08) |
| PZA use | |  |  |  |  |  |  |
|  | No (n = 509) | 179 | 330 |  | 1 (ref) |  | 1 (ref) |
|  | Yes (n = 45) | 11 | 34 |  | 0.60 (0.30–1.21) |  | 0.92 (0.59–1.35) |

Abbreviations: OR, odds ratio; CI, confidence interval; ref, reference; BMI, body mass index;

ADL, activities of daily living; TB, tuberculosis; AST, aspartate aminotransferase; ALT, alanine aminotransferase; CCr, creatinine clearance; RIF, rifampicin; INH, isoniazid; PZA, pyrazinamide.

^*^Concomitant drugs ≥ 7

**Supplementary Figure S1. The estimate of negative sputum culture conversion in the no event group (patients without adverse drug reactions and those who were alive at the 60-day follow-up)**


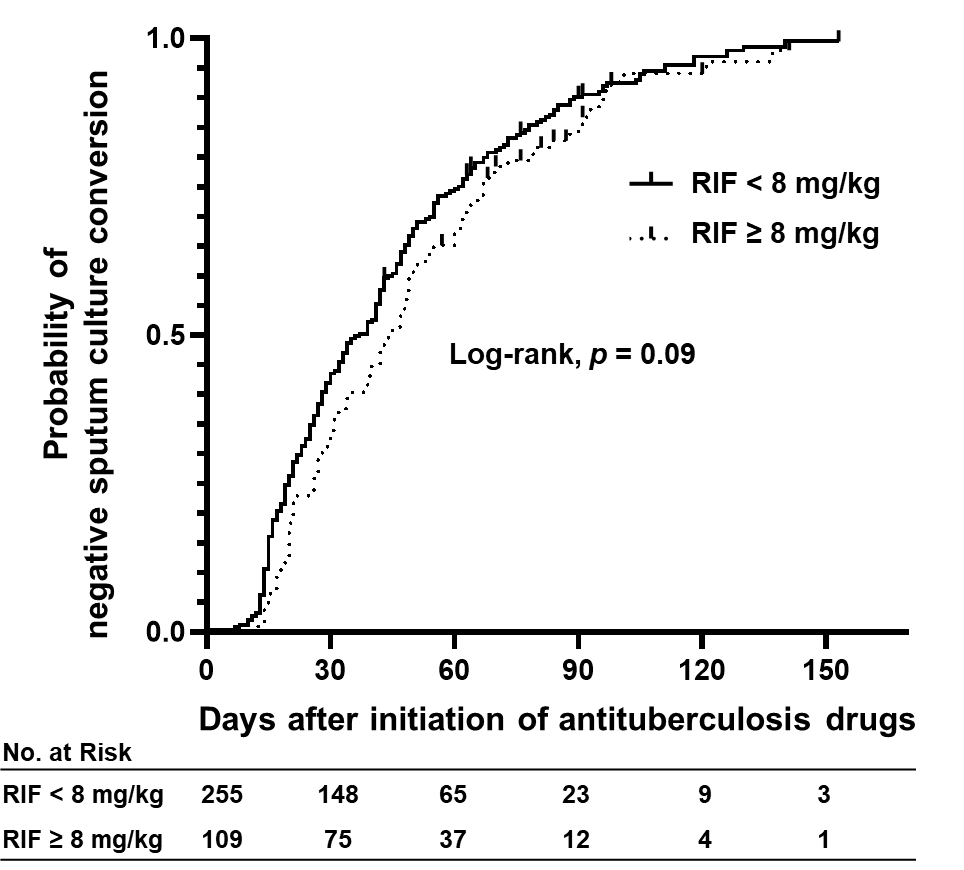


The cumulative incidence of negative sputum culture conversion in the no event group, which included patients without adverse drug reactions who were alive at the 60-day follow-up, was estimated using Kaplan–Meier curves.

Vertical lines indicate censored cases, including patients who were transferred to other hospitals or who died before negative sputum culture conversion could be confirmed.

Abbreviations: RIF, rifampicin; TB, tuberculosis.
